# Core needle biopsy in fibroepithelial tumors: predicting factors for phyllodes tumors

**DOI:** 10.6061/clinics/2021/e2806

**Published:** 2021-04-07

**Authors:** Yedda Nunes Reis, Jonathan Yugo Maesaka, Carlos Shimizu, José Maria Soares-Júnior, Edmund Chada Baracat, José Roberto Filassi

**Affiliations:** I Disciplina de Ginecologia, Departamento de Obstetricia e Ginecologia, Hospital das Clinicas HCFMUSP, Faculdade de Medicina, Universidade de Sao Paulo, Sao Paulo, SP, BR.; II Departamento de Radiologia, Hospital das Clinicas HCFMUSP, Faculdade de Medicina, Universidade de Sao Paulo, Sao Paulo, SP, BR.

**Keywords:** Fibroepithelial Lesions, Phyllodes Tumor, Fibroadenoma, Breast Ultrasound, Core Needle Biopsy

## Abstract

**OBJECTIVES:**

This study aimed to evaluate the clinical and imaging predictive factors for the diagnosis of phyllodes tumors in patients with inconclusive results from core needle biopsy (fibroepithelial lesions).

**METHODS:**

We retrospectively analyzed data of patients who underwent surgical excision of breast lesions previously diagnosed as fibroepithelial lesions. Numeric variables were analyzed using the Shapiro-Wilk and t-tests, and categorical variables were analyzed using the chi-square and Fisher’s exact tests. Multivariate logistic regression was performed to calculate odds ratios and detect predictive factors for the diagnosis of PT.

**RESULTS:**

A total of 89 biopsy samples were obtained from 77 patients, of which 43 were confirmed as fibroadenomas, 43 as phyllodes tumors, and 3 as other benign, non-fibroepithelial breast lesions. The mean tumor size was 3.61 cm (range, 0.8-10 cm) for phyllodes tumors and 2.4 cm (range, 0.8-7.9 cm) for fibroadenomas. The predictive factor for phyllodes tumor diagnosis was lesion size >3 cm (*p*<0.001).

**CONCLUSION:**

Our data indicate that fibroepithelial lesions of the breast larger than 3 cm are more likely to be phyllodes tumors.

## INTRODUCTION

Fibroepithelial tumors of the breast are part of a heterogeneous group of biphasic neoplasms resulting from proliferation of both epithelial and stromal components, which include common fibroadenomas (FA) and phyllodes tumors (PT).

PT is a rare fibroepithelial tumor that is histologically divided into three grades: benign, malignant, or borderline. Although only about 10% of these tumors are malignant, even benign tumors are prone to local recurrence and can become very large in size (1). Given their propensity for local recurrence, the standard management of PTs is surgical excision with negative surgical margins (2).

Despite the use of many pathological parameters, the differential diagnosis of fibroepithelial tumors is often difficult and imprecise with core needle biopsy (CNB), especially because of its heterogeneity and the increased cellularity of the stromal component. In these challenging cases, pathologists are encouraged to describe the lesions as “fibroepithelial lesions” and surgical excision is often suggested to enable a complete evaluation. Identifying predictive factors for the diagnosis of PT in an inconclusive CNB setting will assist the breast surgeon in providing better care for these patients, avoiding over and undertreatment of such lesions (3,4).

This study aimed to evaluate the clinical and sonographic characteristics of patients with inconclusive results in CNB (*i.e.*, fibroepithelial lesions) from a breast biopsy database to explore predictive factors for the diagnosis of PT.

## MATERIALS AND METHODS

The study was approved by the Ethics Committee (approval no. 1.838.324). Informed consent was deemed unnecessary by the Committee, and was therefore not obtained. The search was performed at the Centro de Diagnóstico por Imagem das Doenças da Mama database from the radiology department of the University of São Paulo Medical School General Hospital.

The inclusion criteria were as follows: patients who underwent surgical excision of breast lesions following a CNB result of a fibroepithelial tumor from 2002 to 2013. Patients who did not undergo subsequent excisional biopsy were excluded. Breast ultrasound examinations were retrieved from the University of São Paulo Medical School Department of Radiology database.

CNB was performed under sonographic guidance by dedicated breast radiologists from our service, following standard practices. Excisional biopsies were performed by experienced breast surgeons, and histopathological analyses were performed by general pathologists at our center.

The following variables were analyzed from the collected data: age, clinical presentation (palpable or non-palpable), ultrasonographic characteristics (size, echogenicity, borders, pattern), histopathologic results from CNB, and anatomopathological results from excisional biopsy.

For statistical analysis, we divided the patients into two groups according to the final biopsy result (FA or PT). Patient demographic and clinical characteristics, such as lesion and ultrasound characteristics, were compared to assess potential differences in the two groups.

Numeric variables were analyzed using the Mann-Whitney U test, and categorical variables were analyzed using the chi-square and Fisher’s exact tests. Multivariate logistic regression was performed to calculate odds ratios and detect predictive factors for the diagnosis of PT. The analysis was performed using the SPSS software (version 20.0; IBM SPSS Statistics for Windows, Version 20.0. software; IBM Corp., Armonk, NY, USA).

## RESULTS

### Patient characteristics and management

From the database, we retrieved data on 106 biopsies from 92 patients that were classified as fibroepithelial lesions that could not be further classified as FA or PT between 2002 and 2013. Of these, 17 biopsies from 17 patients were excluded for not having matching excisional biopsies, with a final number of 89 samples suitable for analysis (one patient had four lesions, and five patients had two lesions each). In 12 biopsies, the pathologist added a comment favoring the diagnosis of PT, and in two, the comment favored the diagnosis of FA.

The median age of the patients at diagnosis was 29.7 years (range, 13-55 years) for PT and 28.1 years (range, 19-51 years) for FA. From the available data, 24 patients in each group had palpable lesions. Table 1 summarizes the patients’ demographic characteristics and echocardiographic findings. There were no significant differences in clinical or imaging features.

### Pathologic outcomes

**Figure 1:**
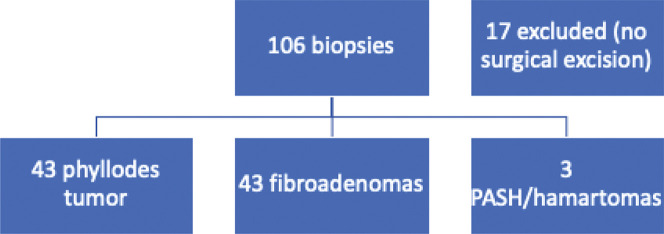
Case selection. PASH: pseudoangiomatous hyperplasia of the mammary stroma.

Of the 89 fibroepithelial lesions excised, the final pathology results were as follows: 43 PT (48.3%), 43 FA (48.3%), 2 (2.2%) pseudoangiomatous hyperplasia of mammary stroma, and 1 (1.2%) hamartoma (Figure 1). In the PT group, four of them were borderline, two were malignant, and the remaining were benign. Our findings for borderline and malignant PTs are summarized in Table 2. In the FA group, three cases were juvenile FA and five were complex.

The median tumor size was 2.95 cm (range, 0.8-10 cm) for PT and 2.1 cm (range, 0.8-7.9 cm) for FA (*p*=0.03). In the multivariate analysis, tumor size ≥3 cm significantly favored the diagnosis of PT (*p*=0.007). In the Fisher’s exact test, tumor size >3 cm was also significant for the diagnosis of PT (odds ratio=4.286, confidence interval=1.515-12.123, *p*=0.007). All other analyzed variables had non-significant *p*-values.

## DISCUSSION

Differentiating PT from FA in the preoperative context is a matter of great importance in clinical practice, since the conservative management of PTs can be worrisome (5,6). Our findings that fibroepithelial tumors larger than 3 cm in CNB have a greater probability of being PTs might be helpful in managing such lesions.

As previously mentioned, tumor size was the single statistically significant finding in differentiating PT and FA in our study, which in line with current medical literature. A retrospective study of 105 patients demonstrated a significant difference in tumor size between PT (4 cm) and FA (2.4 cm) (7). In another study, Yasir et al. also found a difference in tumor size between PT (2.9 cm) and FA (1.8 cm) (8). In a recent cohort that included 141 patients, fibroepithelial lesions in women aged 50 years or older were almost four times more likely to have a PT at surgical excision if the size on the breast ultrasound examination was 2 cm or more (9).

Ultrasonographic findings of nodule orientation, shape, margins, and echogenicity were not statistically significant in our study. Previously published studies have demonstrated controversial results (10), and have shown that the sonographic findings features were unreliable in differentiating FA and PT due to the considerable overlap in findings (9). Otherwise, in a prospectively maintained database study with 48 fibroepithelial tumors excised, PT was more likely to exhibit heterogeneous or complex echotexture than FA (38.9% *vs*. 10%, *p*=0.03) and were less likely to demonstrate internal vascularity. However, no other sonographic characteristics have been reported (11).

An upgrade rate of 48,3% from fibroepithelial lesions for PT was higher than that reported in previous literature, which ranges from 18% to 42% (2,8,12,13). In a large cohort followed over 8 years, 313 fibroepithelial lesions diagnosed in a CNB were divided into two groups: observation and surgical excision. Of the 261 observed patients, only three (1%) were diagnosed with PT on follow-up. Eighteen out of 52 patients (35%) who underwent excision had an upgrade to PT diagnosis (2). It is also worth mentioning the difference in CNB accuracy in diagnosing benign, malignant, and borderline lesions. In 91 patients, Ward et al. found a specificity of 57% for diagnosing benign PTs, 90% for borderline tumors, and 100% for malignant tumors (14).

This study had some limitations. First, this was a retrospective study. In 17 cases (16%), no surgical excision was performed; therefore, we had no surgical biopsies to compare the results of CNB in these cases and they were excluded. Furthermore, we tried to emulate daily practice; thus, the biopsies were not revised by a specialized breast pathologist. In addition, the sample size in our study was limited, and it is possible that a larger number of patients could help identify other factors that might influence the diagnosis of PT.

Our patients were younger than those in the current medical literature, a finding for which we were unable to provide a reasonable explanation; however, this might be related to our small sample size and selection bias (since our service is a tertiary center, younger patients tend to be referred to our center for investigation of breast tumors).

## CONCLUSION

In conclusion, our finding that fibroepithelial lesions larger than 3 cm are more likely to be PT is consistent with the current medical literature. The applicability of this threshold and its implications in clinical practice suggest the need for further studies.

## AUTHOR CONTRIBUTIONS

Maesaka JY, Soares-Júnior JM and Reis YN created the concept and designed the study, collected and analyzed the data, and wrote the manuscript. Baracat EC and Filassi JR reviewed the contents of the manuscript and approved its final version. Reis YN, Maesaka JY, Shimizu C, Soares-Júnior JM, Baracat EC and Filassi JR contributed to the final version of the manuscript.

## Figures and Tables

**Table 1:** Clinical and imaging characteristics of phyllodes tumors and fibroadenomas.

	Phyllodes tumors (n=43)	Fibroadenoma (n=43)	*p*-value>
Age (in years)	29.7 (13-55)	28.1 (19-51)	0.790
Median size (cm)	2.95	2.1	0.03
<3 cm	19	30	0.007
>3 cm	19	7	
Palpable lesion			0.551
Yes	24	24	
No	3	8	
Shape			0.572
Oval/round	28	26	
Lobulated/irregular	7	10	
Orientation			0.516
Parallel	22	28	
Non-parallel	2	2	
Echogenicity			0.418
Hypoechoic	30	34	
Complex	4	2	
Margins			0.866
Circumscribed	29	30	
Poorly defined/Lobulated	6	5	

**Table 2:** Clinical and ultrasonographic characteristics of malignant and borderline phyllodes tumors.

	Case 15	Case 25	Case 30	Case 56	Case 61
Age (years)	55	33	45	36	45
Core biopsy	FEL	FEL	FEL	FEL, PT	FEL, PT
Excisional biopsy	Phyllodes Borderline	Phyllodes Borderline	Phyllodes Malignant	Phyllodes Borderline	Phyllodes Borderline
Palpable	Yes	Yes	Yes	Yes	Yes
Size (mm)	46	68	45	39	100
Echogenicity	Complex	Complex	Complex	Hypoechogenic	Complex
Shape	Irregular	Oval	Irregular	Oval	Oval
Margins	Poorly defined	Circumscribed	Poorly defined	Circumscribed	Circumscribed
Orientations	Non-parallel	Parallel	Parallel	Parallel	Parallel

FEL: fibroepithelial lesion; FEL, PT: fibroepithelial lesions suggesting phyllodes tumor.
